# Preparation and Characterization of Carvacrol-Loaded Caseinate/Zein-Composite Nanoparticles Using the Anti-Solvent Precipitation Method

**DOI:** 10.3390/nano12132189

**Published:** 2022-06-25

**Authors:** Huaming Zheng, Jiangli Wang, Yiqiang Zhang, Quanwei Xv, Qiaohui Zeng, Jingjing Wang

**Affiliations:** 1 School of Material Sciences & Engineering, Wuhan Institute of Technology, Wuhan 430205, China; 22005010049@stu.wit.edu.cn (J.W.); zhangyiqiang_2006@163.com (Y.Z.); xuquanwei929@126.com (Q.X.); 2 Province Key Lab of Plasma Chemistry and Advanced Materials, Wuhan Institute of Technology, Wuhan 430073, China; 3 School of Food Science and Engineering, Foshan University/South China Food Safety Research Center, Foshan 528000, China; baozhuang126@126.com

**Keywords:** zein, carvacrol, sodium caseinate, composite nanoparticles, antibacterial properties

## Abstract

Extending shelf life and maintaining the high quality of food are arduous challenges. In this study, the self-assembly properties of zein were used to load carvacrol essential oil, and then sodium caseinate was selected as a stabilizer to fabricate carvacrol-loaded composite nanoparticles. The results showed that the composite nanoparticles had a high encapsulation efficiency for carvacrol (71.52–80.09%). Scanning electron microscopy (SEM) indicated that the carvacrol-loaded composite nanoparticles were spherical and uniformly distributed, with particle sizes ranging from 80 to 220 nm. First and foremost, the carvacrol-loaded nanoparticles exhibited excellent water-redispersibility, storage-stability, and antioxidant properties, as well as antibacterial properties against *Staphylococcus aureus* and *Escherichia coli*. Benefiting from the antimicrobial and antioxidative abilities, the films with carvacrol-loaded composite nanoparticles effectively inhibited food spoilage and prolonged the shelf-life of cherry tomatoes and bananas. Therefore, carvacrol-loaded composite nanoparticles may have potential application prospects in the food industry.

## 1. Introduction

Numerous safe, active substances extracted from plants have been proven to have good antibacterial, antioxidant, and anticancer effects, but most of the active substances are lipophilic, which limits their application in food and pharmaceutical fields. Therefore, it is necessary to improve the solubility, bioactivity, and bioavailability of lipophilic, active substances through effective methods [1]. Carvacrol (CA), also known as 2-methyl-5-isopropylphenol, is a yellowish-to-yellow, oily, scented compound [2], which is widely found in natural plant essential oils. Due to its excellent antibacterial ability, antioxidant activity, safety, and anti-aging pharmacological effects [3,4], it has attracted more and more researchers’ attention. However, the properties of CA, such as its insolubility in water, volatile nature, and unique odor, limit its application in food preservation [5]. Researchers have improved the stability and sustained release properties of CA by preparing loading systems such as liposomes [6], microcapsules, and emulsions [7], or directly adding CA essential oil into films [8]. By comparing these studies, it was found that reducing the size of the particles to the nanometer level can effectively improve their active-ingredient efficacy, redispersibility, and bioavailability [9].

Zein, an abundant and inexpensive resource, has been generally recognized as a safe food by the US Food and Drug Administration [10]. The zein structure contains a large number of non-polar amino acids [11] and has a hydrophilic top and a hydrophobic outer surface, which exhibits unique solubility properties. It is insoluble in water, but soluble in 60% to 90% aqueous ethanol [12]. Zein has self-assembly properties and biocompatibility [13]. By using the anti-solvent precipitation method [14], the highly polar water environment induces the aggregation of zein ellipsoids to form nanoparticles through hydrophobic interactions. Depending on this property, zein is often used as a delivery system for biologically active ingredients. However, the nanoparticles made from only zein have some disadvantages such as a big diameter, aggregation, and insolubility. Usually, gum arabic (GA), chitosan (CS), tannic acid (TC), and so on were selected as the stabilizer during an anti-solvent procedure [15,16,17]. Sodium caseinate (SC) is the sodium salt of casein, the main protein in milk, and its hydrophobic part can adsorb on zein nanoparticles through hydrophobic interaction. Meanwhile, the hydrophilic part provides spatial and electrostatic repulsion to nanoparticles. Notably, the nanoparticles prepared by this method presented a high redispersibility in water and a high encapsulation efficiency.

The purpose of this study was to form composite nanoparticles that are novel, long-term stable, and redispersible in water, with high encapsulation, by a simple anti-solvent-precipitation method. In addition, the prepared nanoparticles mixed with a PVA film can effectively slow down the rot rate of cherry tomatoes and bananas to prolong their storage life.

## 2. Materials and Methods

### 2.1. Materials

Zein powder with a protein content of 95% (*w*/*w*) was purchased from Beijing Solarbio^®^ Science & Technology, Co. Ltd. (Beijing, China). CA was provided by Marklin (purity ≥ 99%, Shanghai, China). Sodium caseinate (SC) was purchased from Shanghai Tixiai Chemical Industry Development Co., Ltd. (purity ≥ 99%, with the total nitrogen from 14.5 to 15.8% after drying, Shanghai, China). Other reagents were sourced from Sinopharm Chemical Reagent Co., Ltd. (Analytical grade, Shanghai, China). All the solutions used in the experiments were prepared using ultrapure water, which was obtained from a Millipore (Millipore, Milford, MA, USA) Milli-Q water purification system.

### 2.2. Preparation of CA Loaded Composite Nanoparticles

According to Littoz’s method [18], with a slight modification, 0.2 g of zein and 0.1 g of CA were dissolved in 10 mL of 80% (*v*/*v*) aqueous ethanol solutions, and the solutions were stirred with a magnetic stirrer at 45 °C for 2 h until they were completely dissolved. Then, 2 mL of the above solution was added dropwise to 8 mL of ultrapure water and stirred at 400 rpm for 5 min. The residual ethanol in the sample was removed by a rotary evaporation instrument at 40 °C, and ultrapure water was added to the original volume (10 mL) to obtain zein–carvacrol dispersions.

A certain amount of SC was dissolved in 10 mL of ultrapure water, and then the equal volume of above dispersion was dropped into the SC solution, and the pH was adjusted to 6.6. Finally, the CA-loaded composite nanoparticles were formed. In the experiment, the mass ratios of zein/SC were set to be 4:1, 2:1, 1:1, and 1:2 (*w*/*w*), respectively. All samples were freeze-dried at −50 °C for 48 h (Alpha1-4 LD Plus, Christ, Germany). The freeze-dried powders were collected and stored at −20 °C before further use.

### 2.3. Characterization

#### 2.3.1. FT-IR Analysis

Infrared spectra of the samples were obtained by using an FT-IR spectrometer (Nicolet 6700, thermo fisher scientific inc, Waltham, MA, USA). The spectra were recorded over the range of 4000~400 cm^−1^, with a resolution of 4 cm^−1^, by taking 32 scans for each sample. The spectra were analyzed using the OMNIC 8.2 software package (Thermo Fisher Scientific Inc., Waltham, MA, USA).

#### 2.3.2. Measurement of Encapsulation Efficiency (EE)

The measurement of encapsulation efficiency was carried out according to Wang’s method [13], with a slight modification. The standard curve of CA was first established. A certain concentration of CA solution (10 µg/mL~50 µg/mL) was prepared with ethanol as a solvent. UV spectrophotometer (Lambda 35, Perkin Elmer, Waltham, MA, USA) was used to measure the absorbance at a wavelength of 276 nm. Taking the concentration of CA as the abscissa and the absorbance value as the ordinate, the equation of the standard curve is fitted as Y = 0.01439X + 0.0061 (R^2^ = 0.9996).

Afterwards, 4 mL of the fresh composite nanoparticles were mixed with 16 mL of petroleum ether and stirred well for 10 min. Then, 0.5 mL of the upper organic-solvent phase was transferred to a 25 mL reagent bottle, and the bottle was left in a fume hood for 30 min to completely evaporate the petroleum ether. Subsequently, 4 mL of absolute ethanol was added to dissolve CA in the reagent bottle. The absorbance was measured at 276 nm using a UV spectrophotometer (Lambda 35, Perkin Elmer), and ethanol was used as the blank control group. The encapsulation efficiency was then calculated using the following formula:

$$\mathrm{EE}\left(\%\right)=\frac{m_{1}-m_{2}}{m_{1}}\times 100\%$$

where $m_{1}$ represents the total mass of CA in the formula and $m_{2}$ represents the dissociative mass of CA.

#### 2.3.3. Size and Zeta Potential of Composite Nanoparticles

The particle size, average potential, and polydispersity index (PDI) value of CA-loaded composite nanoparticles were measured by a laser particle-size analyzer (Zetasizer Nano-ZS90, Malvern, UK). All samples were diluted with ultrapure water to an appropriate concentration, and the pH was adjusted to 6.6. Each sample was measured in triplicate at 25 °C, and the data were averaged.

#### 2.3.4. Redispersibility of Composite Nanoparticles

A total of 20 mg of lyophilized nanoparticles were dissolved in 10 mL of ultrapure water. After standing for a period, redispersibility was observed.

#### 2.3.5. Storage Stability of Composite Nanoparticles

Briefly, 20 mg of composite nanoparticles with different zein/SC mass ratios were dissolved in 10 mL ultrapure water. The particle size, potential, and PDI values of the dispersions were observed during 15 days of storage.

The release of CA from nanoparticles soluble in water was studied at 25 ℃ and pH 7. Then, 0.1 g freeze-dried zein/SC/CA NPs was dissolved in 50 mL distilled water, and 4 mL dispersions were added into 11 centrifuge tubes after the solution was completely dissolved. At each predetermined time point (1, 4, 9, 18, 21, 24, 36, 45, 48, 56, 60 h), a tube was taken to measure the free CA mass in the dispersion; the detection method is the same as in Section 2.3.2.

#### 2.3.6. Field-Emission Scanning-Electron Microscope (FE-SEM)

The micro-morphology of the CA-loaded composite nanoparticles was observed using a field-emission scanning-electron microscope (Zeiss SIGMA 300, Oberkochen, Germany). The freeze-dried powder was sprinkled evenly on the conductive plate and then sprayed with gold. The experiment of FE-SEM was performed at an operating voltage of 200 kV.

#### 2.3.7. Differential-Scanning Calorimetry (DSC) and Thermal-Gravimetric Analysis (TGA)

DSC was applied to characterize the thermal stability of CA-loaded composite nanoparticles. Experiments were performed by using a differential-scanning calorimeter (DSC25, TA Instrument). Each sample (~5 mg) was sealed in an aluminum box and heated from 25 to 270 °C rate of 10 °C/min. The flow velocity of N_2_ was set at 20 mL/min.

The LE (Loading efficiency) of CA-loaded composite nanoparticles was characterized by thermal-gravimetric analysis on TGA-5500 (TA instruments, Peoria, IL, USA) at a heating rate of 10 °C/min under nitrogen atmosphere. Samples were heated in an aluminum pan from room temperature to 500 °C. The weight-loss percentages were analyzed using Trios 5.1 software. The mass difference between the total weight loss of CA-loaded and blank composite nanoparticles was calculated as LE.

#### 2.3.8. Antioxidant Properties of Composite Nanoparticles

The antioxidant activity of CA-loaded composite nanoparticles was determined by the DPPH method [19]. Then, 3 mL of DPPH-ethanol solution (40 mg/L), and 3 mL of the dispersions of composite nanoparticles with different mass concentrations (0, 60, 80, 100, 120, and 140 µg/mL) were separately added to 10 mL reagent bottle. After mixing, these were incubated with shaking for 1 h at room temperature in the dark. The absorbance of the supernatant was measured at λ = 525 nm using a UV spectrophotometer (Lambda 35, Perkin Elmer). The DPPH-ethanol solution was used as blank control. The antioxidant activity is indexed by the DPPH-clearance rate, and the calculation formula is as follows:

$$\mathrm{DPPH}\left(\%\right)=\frac{A_{0\;}-A_{1}}{A_{0}}\times 100\%$$

where $A_{0}$ represents the absorbance of blank control and $A_{1}$ represents the absorbance of the dispersions of CA-loaded composite nanoparticles.

#### 2.3.9. Evaluation of Antimicrobial Activity

The antimicrobial efficiency of CA-loaded composite nanoparticles against *E. coli* and *S. aureus* was studied by the plate counting method. Briefly, the CA nanoparticles were dissolved in physiological saline with different mass concentrations (0, 2, 6, and 10 mg/mL), which were sterilized under UV lamp irradiation in advance.

*S. aureus* and *E. coli* were, respectively, inoculated into a solid medium and cultured at 37 °C for 24 h. A single colony of *S. aureus* and *E. coli* was selected and cultured in a liquid media for 24 h, then the concentration of bacterial suspension was diluted to 10^8^ CFU/mL. Afterward, 0.1 mL of the diluted bacterial suspension and 1.0 mL of CA-loaded composite nanoparticle dispersions were mixed with an 8.9 mL liquid medium. The mixed liquid underwent continuous shaking for 24 h, and then it was diluted and spread on the medium plates. The composite nanoparticles without CA were used as a control, and the antibacterial efficiency (Y) was calculated as follows:

$$Y=\frac{A-B}{A}\times 100\%$$

where *A* and *B* are the bacterial colonies in the control and CA-loaded composite-nanoparticle samples, respectively.

#### 2.3.10. Assay of CA-Loaded Composite-Nanoparticles Application

Bananas and cherry tomatoes after harvest were wrapped in bags prepared with PVA film or PVA film containing CA-loaded composite nanoparticles, and then they were stored at 23 °C (50% RH humidity) for 15 or 4 days. Fresh bananas and cherry tomatoes without packaging were selected as a negative control. 

## 3. Results and Discussion

### 3.1. FT-IR Analysis

**Figure 1:**
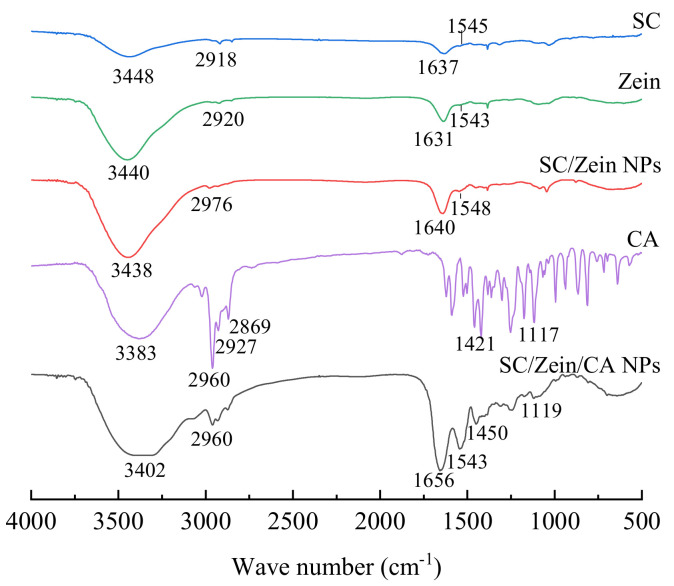
FT-IR spectra of SC, zein, SC/zein NPs, CA, and SC/zein/CA NPs.

The hydrogen bond can be identified by FT-IR analysis. Generally, the larger the frequency (wavenumber) of the infrared-absorption-peak changes, the stronger the hydrogen-bond interaction. FT-IR spectra of each component and the composite nanoparticles are shown in Figure 1. Zein and sodium caseinate showed characteristic peaks at 3440.66 cm^−1^ and 3448.51 cm^−1^, respectively, which were due to the stretching vibration of the hydroxyl group. The infrared spectra of the two proteins showed a similar distribution for the characteristic peaks of amide groups in the region of 1600~1400 cm^−1^ [20]. There is an amide Ⅰ band around 1630 cm^−1^ and an amide Ⅱ band around 1540 cm^−1^, and a total of four absorption peaks were determined in these regions that were attributed to the skeleton vibration of the benzene ring. The absorption peak at 3021.56 cm^−1^ is caused by the unsaturated hydrocarbon’s stretching vibration in the aromatic ring. Generally, the absorption peak of alkyl C-H is a shoulder one, and, therefore, the band of 2960~2869 cm^−1^ is assigned to the alkyl C-H peak. After the zein reacted with the sodium caseinate, the peak of OH shifted to 3438.29 cm^−1^, and it further shifted to 3402.24 cm^−1^ after CA loading. All these facts indicated that strong hydrogen bonds were formed among these substances, owing to the interactions between amide groups in the zein and hydroxyl groups in SC and CA [21]. In zein/SC/CA-composite nanoparticles, a vibration peak was observed at 1450.80 cm^−1^ due to the benzene ring vibration in CA, which further confirmed that CA was successfully embedded in the nanoparticles. The peak at 2920 cm^−1^ corresponded to -CH_3_ vibration in the zein. In the composite nanoparticles, the blue vibration peak of -CH_3_ shifted to 2960.43 cm^−1^, which was caused by the strong hydrophobicity of zein [22]. According to the above results, the stable composite nanoparticles were formed by zein, SC, and CA, which were stabilized by the electrostatic interaction and hydrophobic driving force.

### 3.2. Encapsulation Efficiency of CA-Loaded Composite Nanoparticles

**Figure 2:**
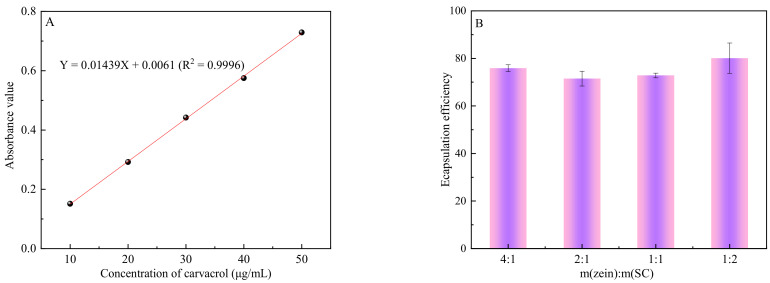
Encapsulation efficiency of nanoparticles with different zein/SC mass ratio. (**A**) The standard curve of CA. (**B**) Encapsulation efficiency of nanoparticles with zein/SC mass ratios of 4:1, 2:1, 1:1, and 1:2.

Encapsulation efficiency is an important characteristic of capsules, which can reflect the degree of drug encapsulation. Firstly, the standard curve of CA was fabricated (Figure 2A). As shown in Figure 2B, the EE of the composite nanoparticles was slightly affected by the zein/SC ratio. On the whole, all treatments in our study had high EE (above 71%), which was comparable to or higher than those of zein-stabilized emulsions using an aqueous-ethanol solution [23,24,25] (in Liu’s study, the EE_max_ is 74.2%; in Gali’s study, the EE_max_ is 69.9% ± 2.3%; and in Chen’s study, the EE_max_ is 26.03% ± 3.20%). In the formula of (m(zein):m(SC) = 4:1), the EE is up to 75.8%. Subsequently, the EE value gradually became bigger with the increase in SC. When the mass ratio of zein to SC was 1:2, the highest EE of the composite nanoparticles reached 80.1%. This may be due to the synergy of zein and SC [13]. On the one hand, the hydrophobic region of SC occupies the internal space of zein particles, which leads to lower EE values. On the other hand, SC forms a thick film on the surface of the zein particles, which reduces the outward diffusion rate of carvacrol. However, the stability and redispersibility of the composite nanoparticles were very poor when the mass ratio of zein/SC was more than 4:1. A proper amount of SC can be used as an electrostatic stabilizer on the surface of the composite nanoparticles, which can prevent the spontaneous aggregation of nanoparticles to form large particles. 

### 3.3. Particle-Size Distribution and Zeta Potential

**Figure 3:**
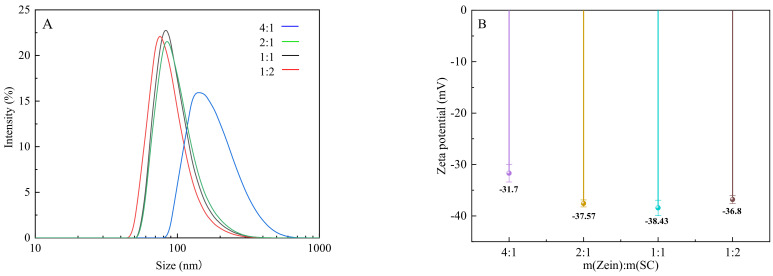
Particle size and zeta potential of the CA-loaded nanoparticles with different zein/SC mass ratios. (**A**) Particle-size distribution. (**B**) the average potential.

In Table 1, the particle size of the composite nanoparticles gradually decreased with the increase in SC content at pH 6.6, and the PDI values of the samples ranged from 0.112 to 0.126. The nanoparticles prepared with a zein/SC mass ratio of 1:2 exhibited the smallest particle size, of 139.6 nm with a narrow distribution. This is because hydrogen bonds are easily formed between zein and SC molecules [26], and hence the interaction between SC and zein in composite nanoparticles becomes stronger with the increase in SC content, which results in reduced particle size. The broad size distribution of the sample with a zein/SC mass ratio of 4:1 might be due to the fact that the amount of SC is not sufficient to saturate the surfaces of the zein nanoparticles and stabilize the nanoparticles. Consequently, the droplets are prone to aggregate (Figure 3A). As is acknowledged, PDI value is usually used to characterize the uniformity of particle-size distribution, and a lower PDI indicates a more uniform particle-size distribution. All samples, except the one prepared with a zein/SC mass ratio of 4:1, have narrow size distributions with small PDI, which reveals that the nanoparticles are monodispersed and stable in water.

The zeta potential of the composite nanoparticles prepared with different zein/SC mass ratios is also shown in Figure 3B. The values of freshly prepared nanoparticles ranged from −31.7 to −38.43 mV. The values are negative because of the carboxylate groups of SC surrounding the particles. Colloidal particles with a zeta-potential magnitude of ≤−35 mV could typically be stabilized by repulsive electrostatic interactions. This may also have been the case for stable dispersions, as observed later in the research. 

### 3.4. Redispersibility of the Composite Nanoparticles after Drying

**Figure 4:**
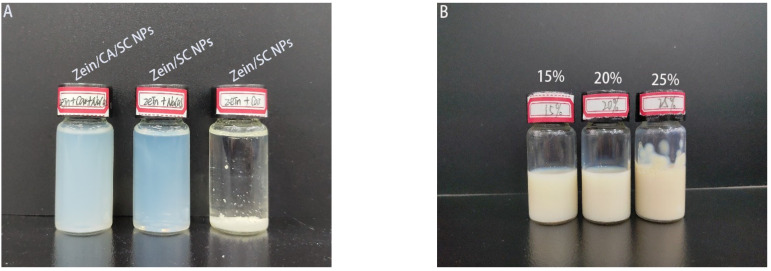
The redispersibility of nanoparticles. (**A**) Different samples at the same mass concentration of 0.2%. (**B**) The redispersibility of zein/CA/SC nanoparticles at mass concentrations of 15%, 20%, and 25%.

The reagent existing in a solid state can be more convenient for transporting than in other states. Redispersibility of the composite nanoparticles after drying is an important characteristic of the encapsulated powder. Figure 4 shows the redispersibility performance of zein/SC/CA nanoparticles, zein/SC nanoparticles, and zein/CA nanoparticles. Obviously, zein/CA nanoparticles without SC showed poor redispersibility, with massive sediments and stratifications at the bottom of the bottle. Nanoparticles with SC possessed an excellent redispersibility, forming a clear and uniform solution, which was consistent with Patel’s report [27]. Furthermore, the prepared zein/SC/CA nanoparticles also presented an excellent redispersibility. When the mass concentration was less than 25%, zein/SC/CA nanoparticles were completely dispersed in ultrapure water, but few visible particles adhered to the wall of the bottle when the mass concentration was above 25%.

### 3.5. Storage Stability of Composite Nanoparticles

**Figure 5:**
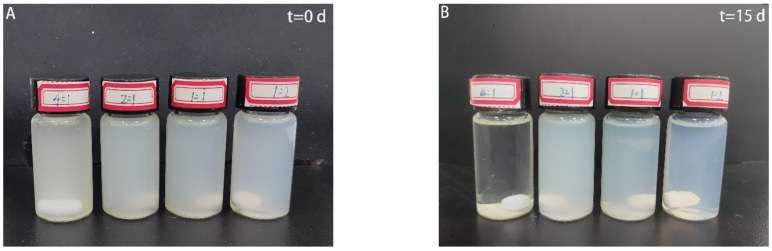
Photographs of CA-loaded nanoparticles with zein/SC mass ratios of 4:1, 2:1, 1:1, and 1:2. (**A**) Freshly prepared. (**B**) Standing after 15 storage days.

The storage stability of CA-loaded nanoparticles with different zein/SC mass ratios (4:1, 1:1, 1:2, and 1:4) was observed in Figure 5. The freshly prepared sample (4:1) was slightly precipitated, and other samples showed good stability at 0 d storage. After standing for 15 days, the composite nanoparticles with a zein/SC mass ratio of 4:1 almost completely precipitated, and those with zein/SC mass ratios of 2:1 and 1:1 displayed a light-precipitation phenomenon. During the whole storage period, only the composite nanoparticles with a zein/SC mass ratio of 1:2 absolutely did not precipitate, and the solution was much clearer and uniform.

**Figure 6:**
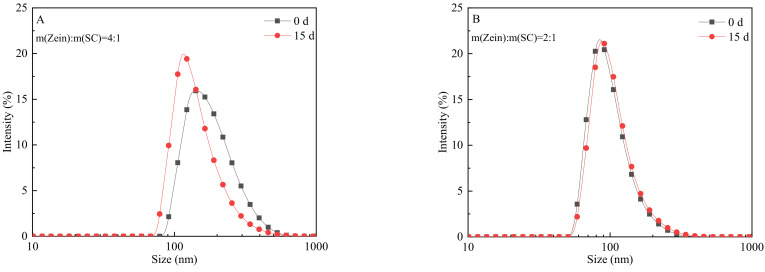

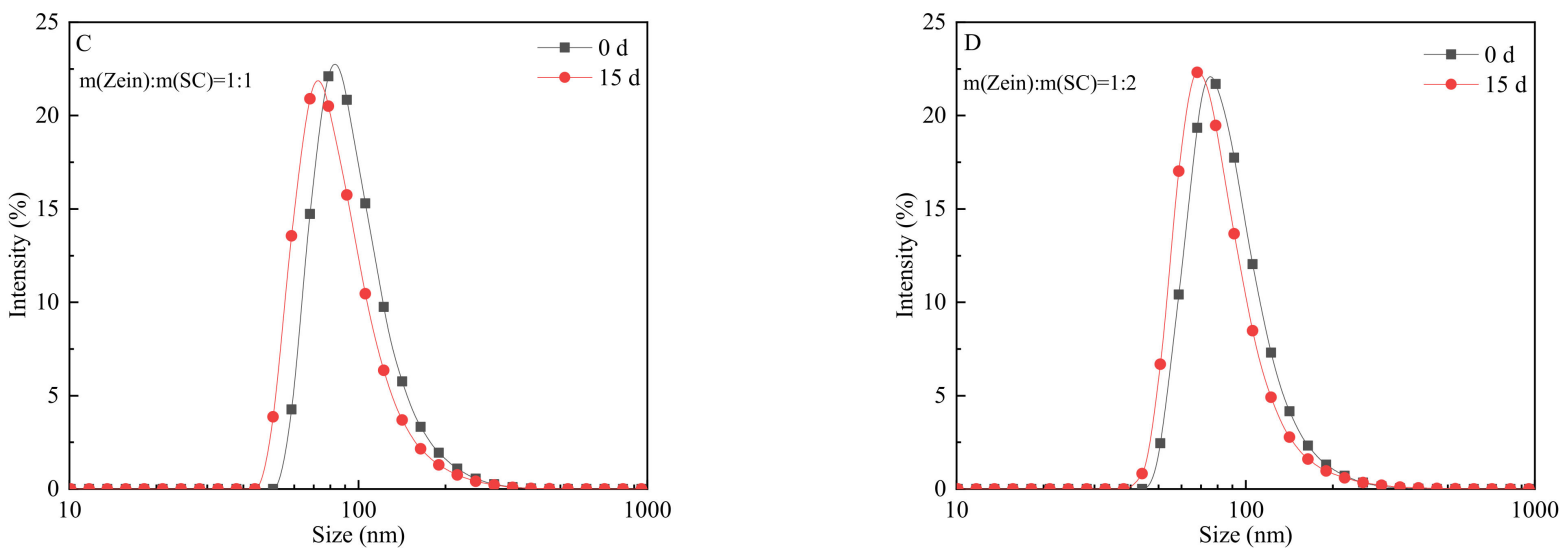
Size distributions of CA-loaded nanoparticles dispersed in water before and after 15 days (different samples with zein/SC mass ratios of (**A**) 4:1, (**B**) 2:1, (**C**) 1:1, and (**D**) 1:2; black square line represents freshly prepared, red spot line represents standing after 15 storage days).

To deeply understand the storage stability of CA-loaded nanoparticles, the particle-size distributions on the 0th day and the 15th day were also analyzed. As shown in Figure 6, it was found that the particle-size distribution of the sample with a zein/SC mass ratio of 4:1 significantly decreased after standing for 15 d, while the others did not obviously change. The main reason might be a little SC is not enough to stabilize the droplets, so, as a result, there are some droplet aggregations and precipitates. The particles in the supernatant are much smaller with a narrow distribution peak.

**Figure 7:**
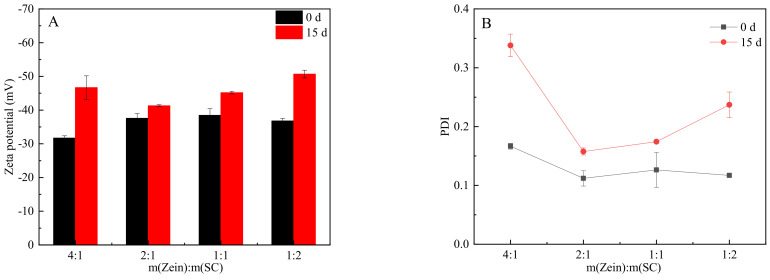
Zeta potential and PDI of CA-loaded nanoparticles with zein/SC mass ratios of 4:1, 2:1, 1:1, and 1:2. (**A**) The zeta-average potential. (**B**) PDI (black represents freshly prepared; red represents standing after 15 storage days).

Furthermore, the zeta potential of all dispersions greatly increased after standing for 15 d, and the maximum zeta potential value of the composite nanoparticles with m(zein): m(SC) = 1:2 reached −50.7 mV (Figure 7A), indicating that these had a superior storage stability compared with the others. By comparing the PDI of all dispersions on the 0th day and the 15th day (Figure 7B), the uniformity of the composite nanoparticles deteriorated during the standing process, owing to the agglomeration of some nanoparticles. In conclusion, the composite nanoparticles with a mass ratio of 1:2 possessed the best storage stability, while the composite nanoparticles (m(zein): m(SC) = 4:1) could hardly form dispersions. 

**Figure 8:**
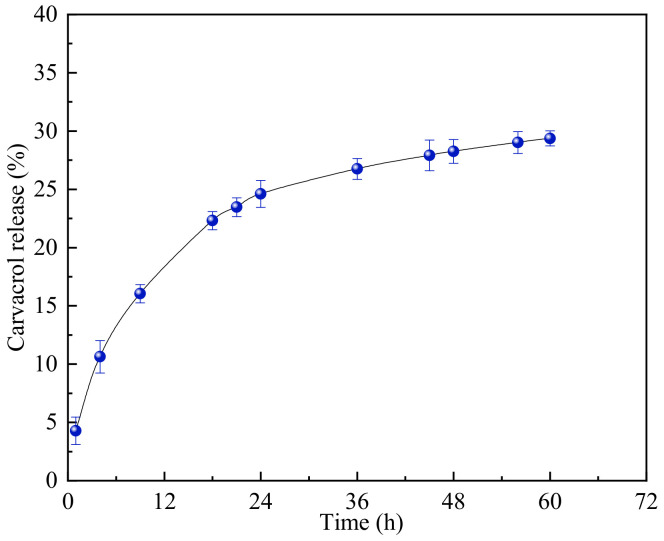
The release of CA from zein/SC/CA nanoparticles soluble in water at 25 °C and pH 7.

The release rate of the active substances in the composite nanoparticles mainly depends on many factors, such as the pH of the solution, the hydrophilicity and size of the nanoparticles, the active substances’ loading capacity [28], etc. A good carrier system can make the encapsulated active substances be released continuously for a period of time. By analyzing the release of CA from zein/SC/CA NPs soluble in water at 25 °C and pH 7 (Figure 8), it can be found that the release process of CA is mainly divided into two stages: rapid release (1–24 h) followed by slow and lasting release (24–60 h). In the initial stage, the cumulative release amounts of CA at 4 h, 9 h, 18 h, and 24 h were 10.63%, 16.04%, 22.31%, and 24.61%, respectively. In the slow-release stage, the cumulative release amounts of CA at 36 h, 48 h, and 60 h were 26.75%, 28.26%, and 29.37%, respectively. These results indicate that the nanoparticles formed by the interaction of zein and SC can effectively slow down the release rate of carvacrol and improve the stability of CA.

### 3.6. FE-SEM Analysis

**Figure 9:**
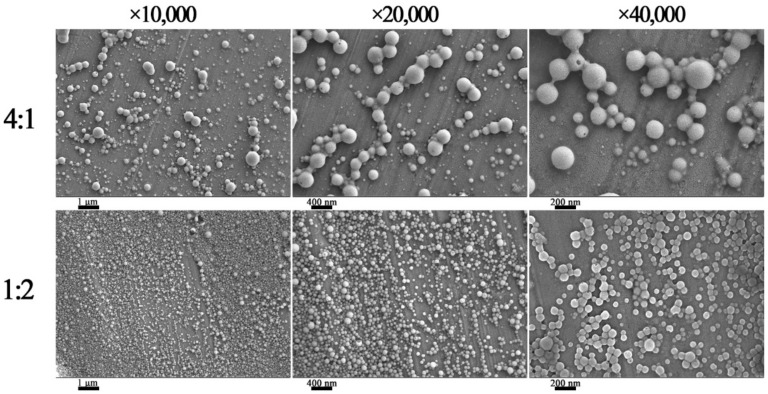
FE-SEM image of CA-loaded nanoparticles prepared with a zein/SC mass ratio of 4:1 (**above**) and 1:2 (**below**).

The microstructure of the composite nanoparticles was characterized by FE-SEM. In Figure 9, the CA-loaded composite nanoparticles displayed a spherical structure [29]. The particle size of the composite nanoparticles with m(zein):m(SC) = 1:2 was about 80~120 nm, and the nanoparticles were evenly distributed. This was mainly because a proper amount of SC interacted with proteins, forming negatively charged COO^−^ on the surface of the particles, which increased the electrostatic repulsion to prevent aggregation. However, the particle size of the composite nanoparticles with m(zein):m (SC) = 4:1 was uneven, with values of ~240 nm. Such a phenomenon was greatly attributed to the absence of SC, which led to the instability of the composite nanoparticles. Then, their agglomeration formed large particles.

### 3.7. DSC and TGA Analysis

**Figure 10:**
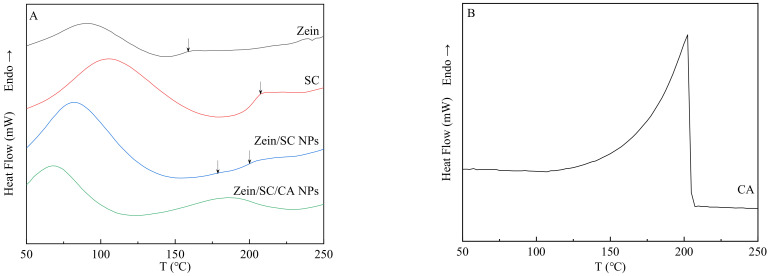
DSC curves of (**A**) zein, SC, zein/SC NPs, and zein/SC/CA NPs. (**B**) Pure CA.

DSC was carried out to characterize the thermal properties of samples [30]. DSC of zein, SC, zein/SC NPs, and zein/SC/CA NPs are shown in Figure 10A. Considering the volatile nature of CA, all samples were subjected to a single heating. The wide endothermic peaks of zein and SC at about 90 °C and 105 °C, respectively, are mainly caused by the evaporation of bound water in the samples. However, the endothermic peaks of zein/SC NPs and zein/SC/CA NPs appear at about 82 °C and 67 °C, respectively, which may be due to the free water. In addition, it is easier for the free water to evaporate. T_g_ values of zein and SC were about 160 °C and 207 °C, respectively, which was consistent with Pereira’s report [31]. In the zein/SC NPs, there are two T_g_ values (177 °C and 199 °C). The possible reason is that weak hydrogen and hydrophobic bonds between zein and SC occur during composite-nanoparticle formation. Compared with the blank nanoparticles, zein/SC/CA NPs showed a new absorption peak at about 180 °C, which is mainly caused by the evaporation of CA. In Figure 10B, a strong absorption peak appeared from 150 °C to 200 °C, which was caused by the evaporation of CA. So, it can be confirmed that CA is successfully embedded in the nanoparticles.

**Figure 11:**
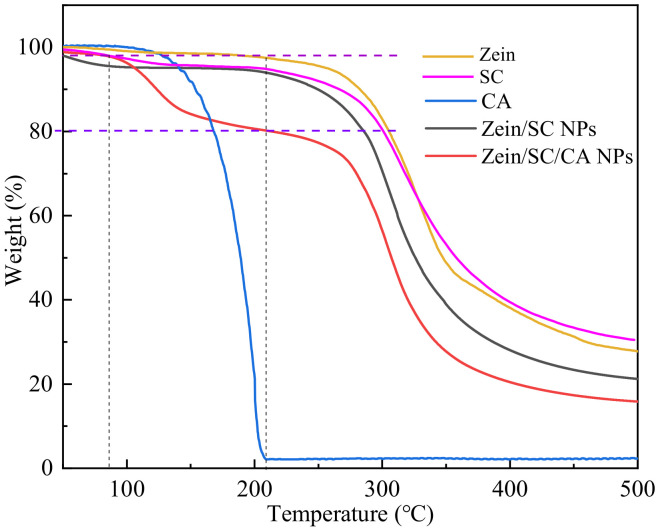
TGA curves of zein, SC, CA, zein/SC NPs, and zein/SC/CA NPs.

In this study, TGA method was employed to further verify the loading efficiency of CA-loaded composite nanoparticles. Figure 11 illustrates the TGA curve of zein, SC, CA, CA-loaded, and blank composite nanoparticles. The mass loss of zein and SC was 1.36% and 4.01%, respectively, at about 127 °C, mainly due to the presence of a small amount of water in the two substances [32]; in addition, from 127 °C to 200 °C, zein and SC have almost no mass loss. From 200 °C to 400 °C, both zein and SC showed a large mass loss of 59.14% and 57.75%, respectively. This is mainly due to the decomposition of the amino acids and peptide bonds in proteins [33]. At 500 °C, the mass of zein and SC is only 27.49% and 30.29%, respectively, which may be the thermally stable residue C_X_H_Y_N_Z_ under nitrogen. These results show that zein and SC are almost stable below ~200 °C. When CA was heated to about 210 °C, it was absolutely volatilized. The blank zein/SC nanoparticles was taken as a control, and about 18% mass loss was regarded as CA-loading efficiency in the zein/SC/CA nanoparticles.

### 3.8. Oxidation-Resistance Property

**Figure 12:**
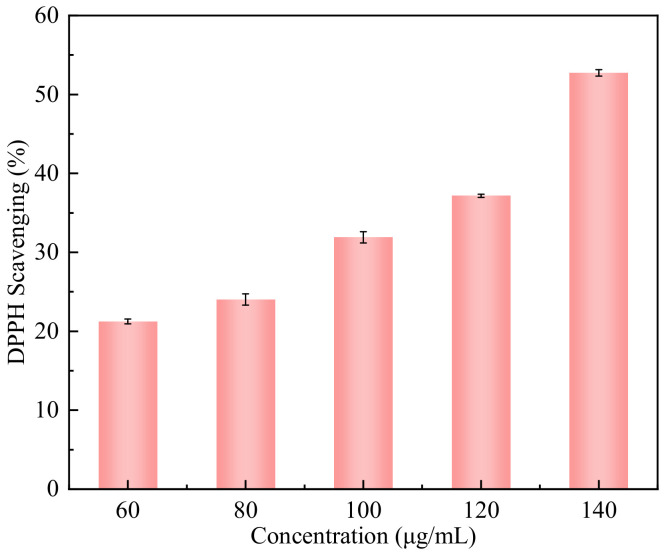
DPPH-scavenging rates of CA-loaded composite nanoparticles with different mass concentrations.

The DPPH free-radical-scavenging rate was used to investigate the antioxidant activity of CA-loaded composite nanoparticles. As illustrated in Figure 12, the antioxidant capacity of CA-loaded composite nanoparticles significantly increased, reaching a maximum of about 54% with an increase in their mass concentration from 60 μg/mL to 140 μg/mL. When the mass concentration of the nanoparticles was 80 μg/mL, the corresponding DPPH scavenging was about 25%. The results showed that the prepared CA-loaded composite nanoparticles had outstanding antioxidant activity at the appropriate mass concentration, and the higher the content of CA was, the better the antioxidant activity of the nanoparticles [34].

### 3.9. Antimicrobial Activity

**Figure 13:**
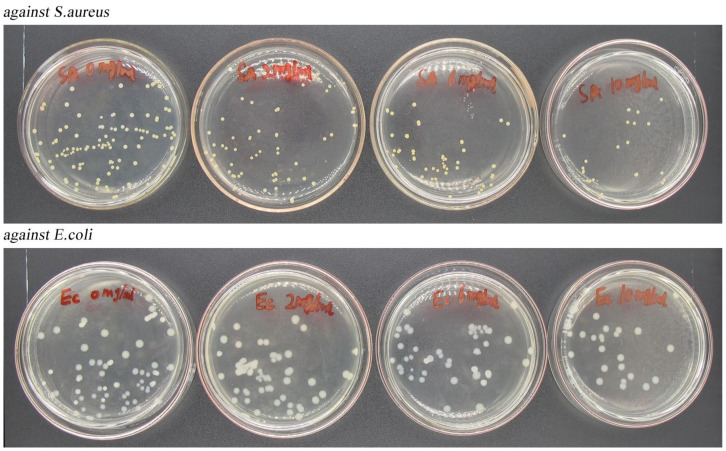
Antibacterial efficiency of CA-loaded composite nanoparticles against *S. aureus* and *E. coli* (from left to right, the mass concentration of nanoparticles is 0%, 2%, 6%, and 10%).

The antimicrobial activity of the CA-loaded composite nanoparticles against *S. aureus* and *E. coli* is demonstrated in Figure 13. As the content of nanoparticles increased, the number of viable colonies gradually decreased, indicating that the CA-loaded composite nanoparticles exhibited a certain antimicrobial efficiency on *S. aureus* and *E. coli*. When the mass concentration of nanoparticles were 2 mg/mL, 6 mg/mL and 10 mg/mL, the antibacterial rates were 40%, 66%, and 80% against *S. aureus* and 33%, 40%, and 66.7% against *E. coli*, respectively. Notably, the CA-loaded composite nanoparticles were more sensitive to *S. aureus* than *E. coli*.

### 3.10. Assay of CA-Loaded Composite-Nanoparticles Application in Food

**Figure 14:**
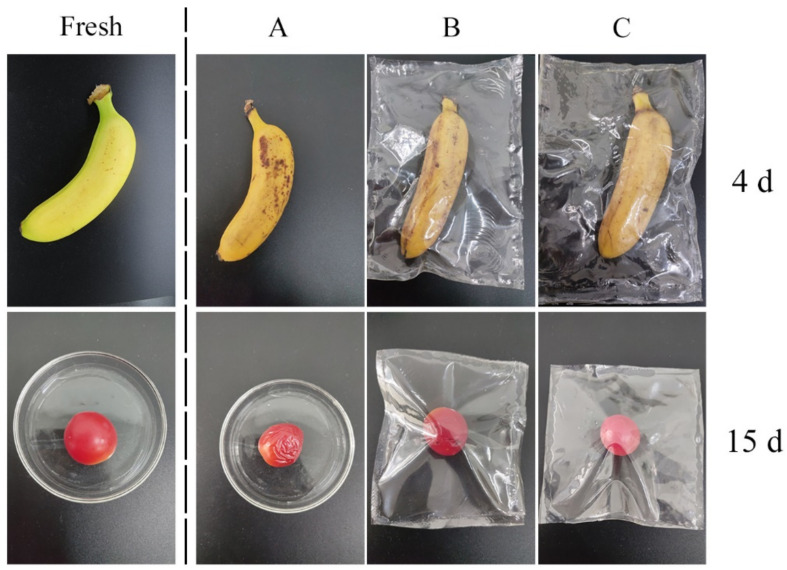
Assay of CA-loaded composite nanoparticles application in bananas and cherry tomatoes (left, fresh bananas and cherry tomatoes; (**A**) storage in air; (**B**) wrapped in PVA film; (**C**) wrapped in PVA film containing 0.25 wt% CA-loaded composite nanoparticles).

**Figure 15:**
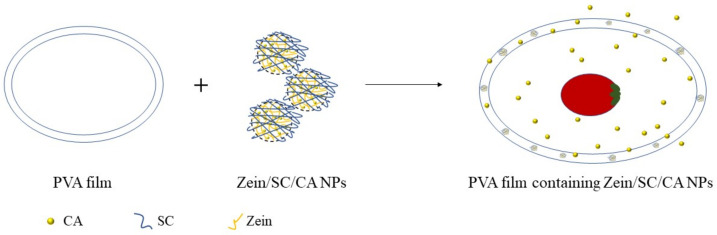
Schematic illustration of films containing CA-loaded composite nanoparticles for keeping food fresh.

It is well-known that bacteria and oxygen are the main causes of food spoilage. To verify the ability of CA-loaded composite nanoparticles to keep food fresh, PVA films and PVA films containing CA-loaded composite nanoparticles were fabricated. Bananas and cherry tomatoes were, respectively, wrapped in those films and stored at 23 °C with a relative humidity of 50%. In Figure 14, the fresh bananas and cherry tomatoes were brightly colored with a rigid texture (left). For banana samples, there were mostly brown spots on the surface when they were stored in air for 4 days. However, the brown spots became smaller when they were sealed in film. Especially in the PVA films containing CA-loaded composite nanoparticles, the brown spots were barely observed because the CA-loaded composite nanoparticles do have an antioxidative ability. The cherry tomatoes were severely decomposed after 15 days without any packaging films. The ones wrapped in the PVA films had some mildew and became soft. However, the cherry tomatoes wrapped in the PVA films containing CA-loaded composite nanoparticles maintained the preferable color and texture as a result of the antibacterial effect of the CA-loaded composite nanoparticles (Figure 15). In addition, some scholars have found that the film-matrix containing CA also has an obvious antioxidative and antibacterial effect on chicken, meat, beef, and so on [35,36,37]. Therefore, it is concluded that CA-loaded composite nanoparticles have great potential to be applied for food preservation.

## 4. Conclusions

In this study, the CA-loaded composite nanoparticles were prepared by the reverse-solvent-precipitation method. When the mass ratio of zein/SC was 1:2, the composite nanoparticles had a narrow particle-size distribution, high encapsulation efficiency, excellent redispersibility, and storage stability after drying and possessed outstanding antioxidant and antibacterial properties. The CA-loaded composite nanoparticles would have a broad application prospect in food preservation and shelf-life extension.

## Figures and Tables

**Table 1:** Particle size and PDI values of the CA-loaded nanoparticles with different zein/SC mass ratios.

Zein/SC Mass Ratios	4:1	2:1	1:1	1:2
Particle size/nm	235.6 ± 5	150.6 ± 0.5	148.8 ± 2.5	139.6 ± 2.3
PDI	0.17 ± 0.005	0.112 ± 0.01	0.126 ± 0.02	0.117 ± 0.004

Note: Values are expressed as mean ± standard deviation (*n* = 3).

## Data Availability

The data presented in this study are available in this article.

## References

[1] Yang J., Wang D., Feng L., Li D.P., Huang Q.R. (2020). Cinnamon essential oil Pickering emulsion stabilized by zein-pectin composite nanoparticles: Characterization, antimicrobial effect and advantages in storage application. Int. J. Biol. Macromol..

[2] Liu F., Jin P.P., Sun Z.L., Du L.H., Wang D.Y., Zhao T., Doyle M.P. (2021). Carvacrol oil inhibits biofilm formation and exopolysaccharide production of enterobacter cloacae. Food Control.

[3] Donsì F., Ferrari G. (2016). Essential oil nanoemulsions as antimicrobial agents in food. J. Biotechnol..

[4] Figueroa-Lopez K.J., Torres-Giner S., Enescu D., Cabedo L., Cerqueira M.A., Pastrana L.M., Lagaron J.M. (2020). Electrospun active biopapers of food waste derived poly(3-hydroxybutyrate-co-3-hydroxyvalerate) with short-term and long-term antimicrobial performance. Nanomaterials.

[5] Silva F.T.D., Cunha K.F.D., Fonseca L.M., Antunes M.D., Halal S.L.M.E., Fiorentin Â.M., Zavareze E.D.R., Dias A.R.G. (2018). Action of ginger essential oil (Zingiber officinale) encapsulated in proteins ultrafine fibers on the antimicrobial control in situ. Int. J. Biol. Macromol..

[6] de Carvalho S.M., Noronha C.M., Floriani C.L., Lino R.C., Rocha G., Bellettini I.C., Ogliari P.J., Barreto P.L.M. (2013). Optimization of α-tocopherol loaded solid lipid nanoparticles by central composite design. Ind. Crop. Prod..

[7] Motta Felício I., Limongi De Souza R., Oliveira Melo C., Gervázio Lima K.Y., Vasconcelos U., Olímpio De Moura R., Eleamen Oliveira E. (2021). Development and characterization of a carvacrol nanoemulsion and evaluation of its antimicrobial activity against selected food-related pathogens. Lett. Appl. Microbiol..

[8] Xu T., Gao C.C., Yang Y.L., Shen X.C., Huang M.G., Liu S.W., Tang X.Z. (2018). Retention and release properties of cinnamon essential oil in antimicrobial films based on chitosan and gum arabic. Food Hydrocoll..

[9] Yen F.L., Wu Z.H., Zeng C.W., Lin L., Lin C. (2010). Curcumin nanoparticles improve the physicochemical properties of curcumin and effectively enhance its antioxidant and antihepatoma activities. J. Agric. Food Chem..

[10] Weissmueller N.T., Lu H.D., Hurley A., Prud Homme R.K. (2016). Nanocarriers from GRAS zein proteins to encapsulate hydrophobic actives. Biomacromolecules.

[11] Feng S.M., Sun Y.X., Wang D., Sun P.L., Shao P. (2020). Effect of adjusting pH and chondroitin sulfate on the formation of curcumin-zein nanoparticles: Synthesis, characterization and morphology. Carbohyd. Polym..

[12] Dai L., Wei Y., Sun C.X., Mao L., McClements D.J., Gao Y.X. (2018). Development of protein-polysaccharide-surfactant ternary complex particles as delivery vehicles for curcumin. Food Hydrocoll..

[13] Wang L., Zhang Y. (2017). Eugenol nanoemulsion stabilized with zein and sodium caseinate by self-assembly. J. Agric. Food Chem..

[14] Kringel D.H., Silva W.M.F., Biduski B., Waller S.B., Lim L.T., Dias A.R.G., Zavareze E.D.R. (2020). Free and encapsulated orange essential oil into a β-cyclodextrin inclusion complex and zein to delay fungal spoilage in cakes. J. Food Process. Pres..

[15] Dai L., Sun C.X., Wei Y., Mao L.K., Gao Y.X. (2018). Characterization of Pickering emulsion gels stabilized by zein/gum arabic complex colloidal nanoparticles. Food Hydrocoll..

[16] Hu S.Q., Wang T.R., Fernandez M.L., Luo Y.C. (2016). Development of tannic acid cross-linked hollow zein nanoparticles as potential oral delivery vehicles for curcumin. Food Hydrocoll..

[17] Liang J., Yan H., Wang X.L., Zhou Y.B., Gao X.L., Puligundla P., Wan X.C. (2017). Encapsulation of epigallocatechin gallate in zein/chitosan nanoparticles for controlled applications in food systems. Food Chem..

[18] Littoz F., McClements D.J. (2008). Bio-mimetic approach to improving emulsion stability: Cross-linking adsorbed beet pectin layers using laccase. Food Hydrocoll..

[19] Hamelian M., Varmira K., Veisi H. (2018). Green synthesis and characterizations of gold nanoparticles using thyme and survey cytotoxic effect, antibacterial and antioxidant potential. J. Photochem. Photobiol. B Biol..

[20] Chang C., Wang T.R., Hu Q.B., Yang C. (2017). Zein/caseinate/pectin complex nanoparticles: Formation and characterization. Int. J. Biol. Macromol..

[21] Veneranda M., Hu Q., Wang T., Luo Y., Castro K., Madariaga J.M. (2018). Formation and characterization of zein-caseinate-pectin complex nanoparticles for encapsulation of eugenol. LWT-Food Sci. Technol..

[22] Zhang H., Fu Y., Xu Y.J., Niu F.G., Li Z.Y., Ba C.J., Jin B., Chen G.W., Li X.M. (2019). One-step assembly of zein/caseinate/alginate nanoparticles for encapsulation and improved bioaccessibility of propolis. Food Funct..

[23] Liu Y.X., Liang Q.F., Liu X.Q., Raza H., Ma H., Ren X.F. (2022). Treatment with ultrasound improves the encapsulation efficiency of resveratrol in zein-gum arabic complex coacervates. Food Sci. Technol..

[24] Gali L., Bedjou F., Ferrari G., Donsì F. (2022). Formulation and characterization of zein/gum arabic nanoparticles for the encapsulation of a rutin-rich extract from ruta chalepensis. Food Chem..

[25] Chen H.Q., Zhang Y., Zhong Q.X. (2015). Physical and antimicrobial properties of spray-dried zein–casein nanocapsules with co-encapsulated eugenol and thymol. J. Food Eng..

[26] Luis A.I.S., Campos E.V.R., de Oliveira J.L., Guilger-Casagrande M., de Lima R., Castanha R.F., de Castro V.L.S.S., Fraceto L.F. (2020). Zein nanoparticles impregnated with eugenol and garlic essential oils for treating fish pathogens. ACS Omega.

[27] Patel A.R., Bouwens E.C., Velikov K.P. (2010). Sodium caseinate stabilized zein colloidal particles. J. Agric. Food Chem..

[28] Pourhossein A., Rafizadeh M., Chen P. (2020). Stimuli-responsive zein-based nanoparticles as a potential carrier for ellipticine: Synthesis, release, and in vitro delivery. Polym. Advan. Technol..

[29] Beirão-da-Costa S., Duarte C., Bourbon A.I., Pinheiro A.C., Januário M.I.N., Vicente A.A., Beirão-da-Costa M.L., Delgadillo I. (2013). Inulin potential for encapsulation and controlled delivery of oregano essential oil. Food Hydrocoll..

[30] Torres-Giner S., Lagaron J.M. (2010). Zein-based ultrathin fibers containing ceramic nanofillers obtained by electrospinning. I. Morphology and thermal properties. J. Appl. Polym. Sci..

[31] Pereira R.N., Souza B.W.S., Cerqueira M.A., Teixeira J.A., Vicente A.A. (2010). Effects of electric fields on protein unfolding and aggregation: Influence on edible films formation. Biomacromolecules.

[32] Mocanu A.M., Moldoveanu C., Odochian L., Paius C.M., Apostolescu N., Neculau R. (2012). Study on the thermal behavior of casein under nitrogen and air atmosphere by means of the TG-FTIR technique. Thermochim. Acta.

[33] Rodríguez-Félix F., Del-Toro-Sánchez C.L., Tapia-Hernández J.A. (2020). A new design for obtaining of white zein micro- and nanoparticles powder: Antisolvent-dialysis method. Food Sci. Biotechnol..

[34] Shao Y., Wu C.H., Wu T.T., Li Y., Chen S.G., Yuan C.H., Hu Y.Q. (2018). Eugenol-chitosan nanoemulsions by ultrasound-mediated emulsification: Formulation, characterization and antimicrobial activity. Carbohyd. Polym..

[35] Wang L., Heising J., Fogliano V., Dekker M. (2020). Fat content and storage conditions are key factors on the partitioning and activity of carvacrol in antimicrobial packaging. Food Packag. Shelf Life.

[36] Sara A.B., Mattijs J.F., Henk P.H., Frans V.K., Edwin J.A.V. (2007). Inhibition of Salmonella enterica serotype Enteritidis on agar and raw chicken by carvacrol vapour. Int. J. Food Microbiol..

[37] He W.Q., Wang Z.Y., Hou C.S., Huang X., Yi B., Yang Y., Zheng W.R., Zhao X., Yao X. (2020). Mucus-inspired supramolecular adhesives with oil-regulated molecular configurations and long-lasting antibacterial properties. ACS Appl. Mater. Interfaces.

